# Supplementary figures and images for: The auxin phenylacetic acid induces NIN expression in the actinorhizal plant Datisca glomerata, whereas cytokinin acts antagonistically
Source: PLoS One. 2025 Feb 3;20(2):e0315798. doi: 10.1371/journal.pone.0315798 (PMC11790169; doi:10.1371/journal.pone.0315798)

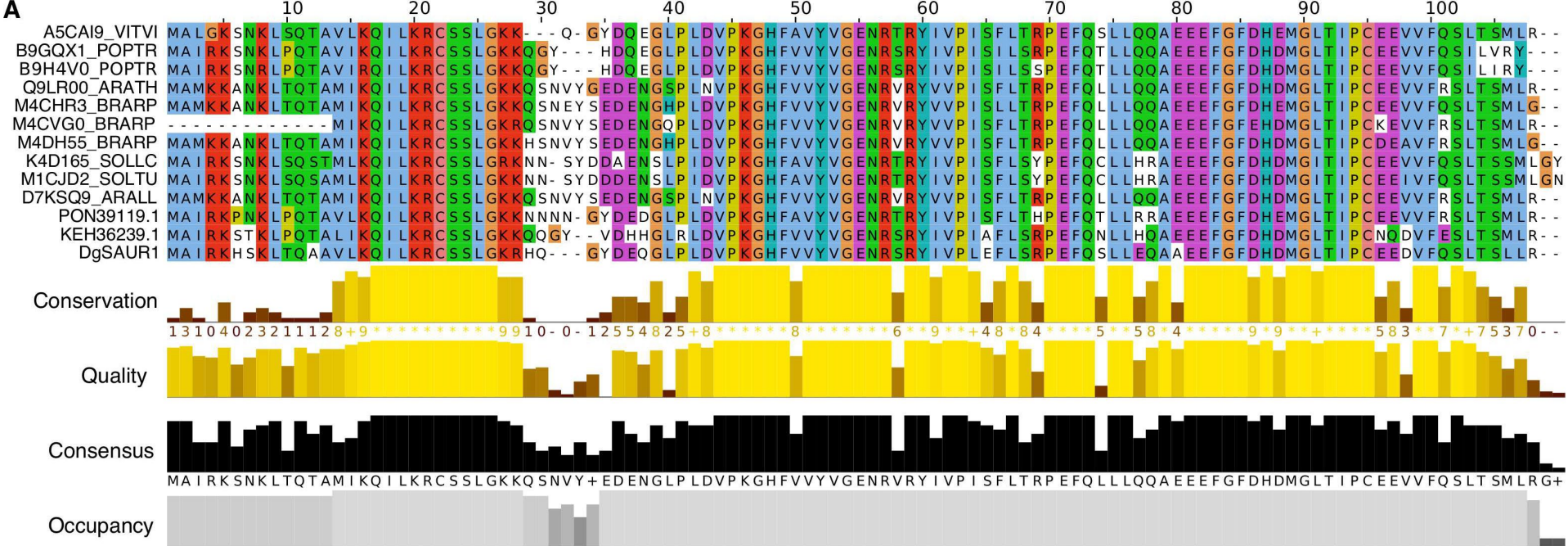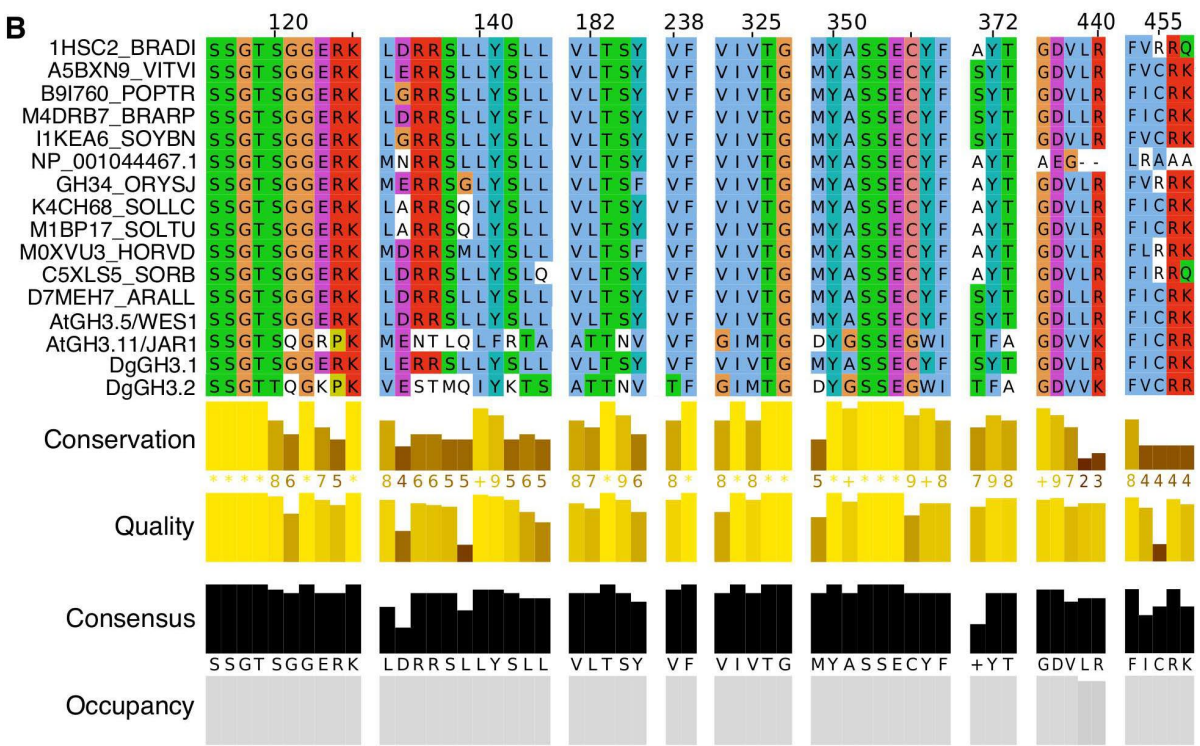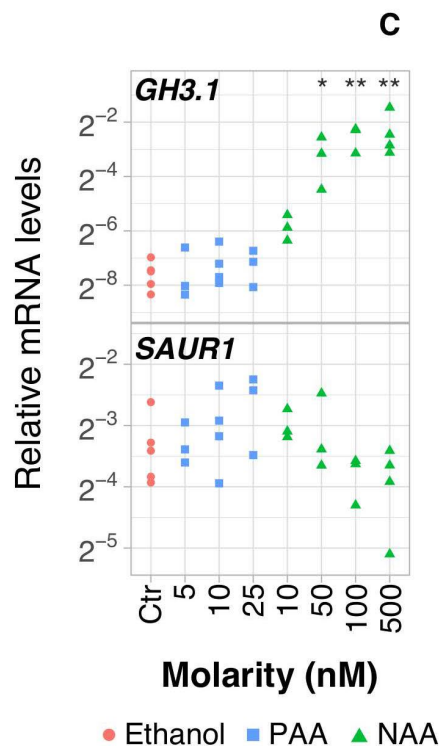

Supplement: S1 Fig — (A) A member of the Small Auxin-Up RNA family of D. glomerata (DgSAUR1) shows high sequence similarity with members of the orthologous group ENOG410J0BD along with proteins from Parasponia andersonii (PON39119.1) and Medicago truncatula (KEH36239.1). (B) Members of the Gretchen Hagen 3 family are expressed in nodules of D. glomerata; DgGH3.1 and DgGH3.2 were compared by multiple sequence alignment with twelve sequences from the orthologous group ENOG410IHQ2 (B). To this set, previously characterized sequences from Arabidopsis thaliana were added: AtGH3.5/WES1 and AtGH3.1/JAR1. While AtGH3.5/WES1 has high affinity for both indole-3-acetic and salicylic acid (Westfall et al., 2016), AtGH3.11/JAR1 responds to jasmonic acid (Westfall et al., 2012). Illustrated blocks represent individual motifs to which a link of structural arrangement with function has been demonstrated (refer to Figures 2 and 3 of Westfall et al., 2016). Additional metrics like conservation, quality, consensus, and occupancy are given. (C) Genetic dependencies of these promoters to auxins on 54-day-old D. glomerata roots. Y-axis shows the mRNA quantity relative to that of PUQ, EF1-α, and TIP41. Phytohormones and molarities are given on the X-axis. Significant differences to the control are highlighted by Welch’s pairwise t-test with Holm’s correction at p<0.01 (**). Gene names are given. (PDF) [file pone.0315798.s001.pdf]

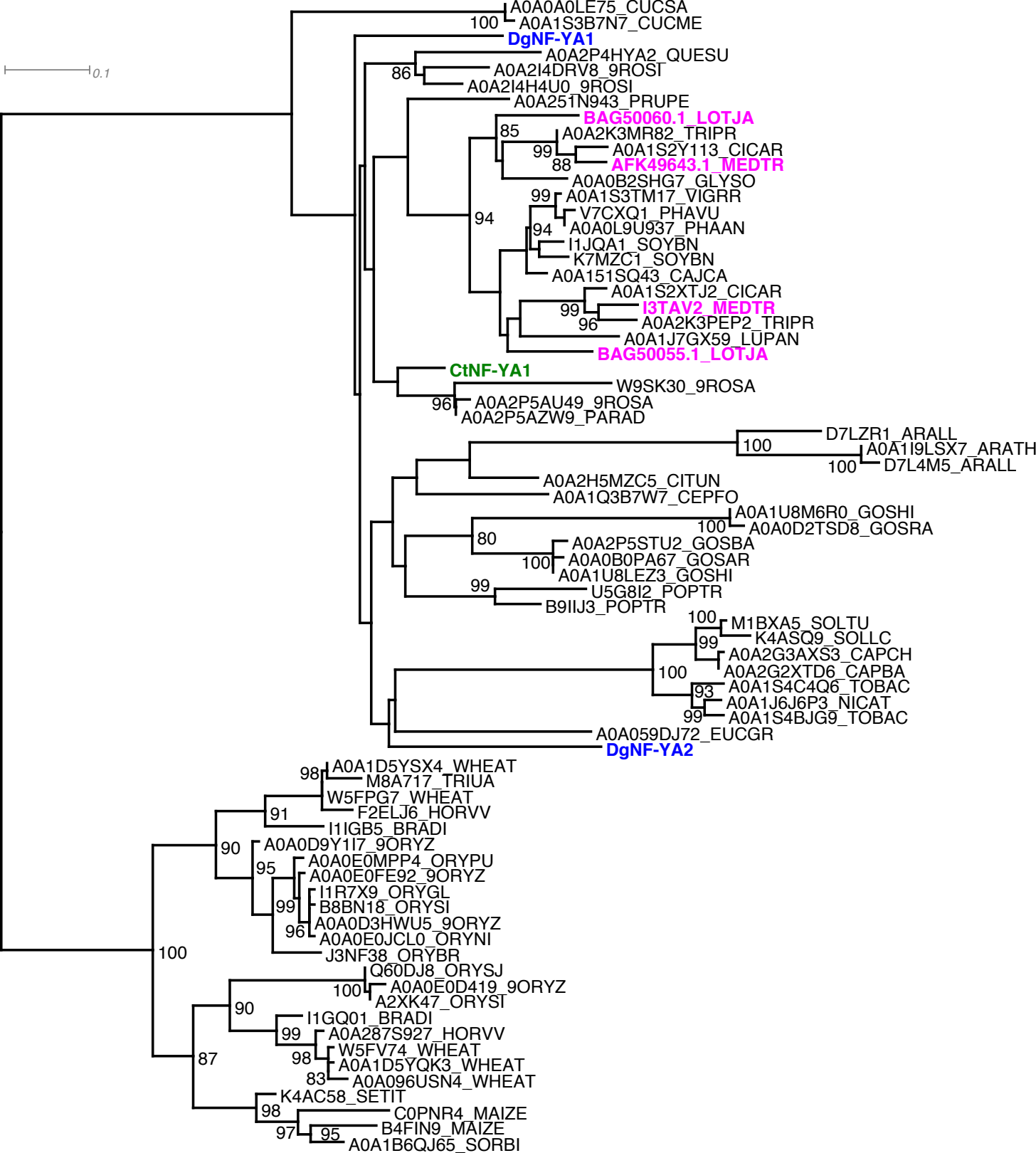

Supplement: S3 Fig — Nomenclature is from UniProtKb. Proteins from model legumes were highlighted in pink, Ceanothus thyrsiflorus in green (GenBank accession MN388814), and Datisca glomerata in blue. (PDF) [file pone.0315798.s003.pdf]

Fold change in mRNA levels

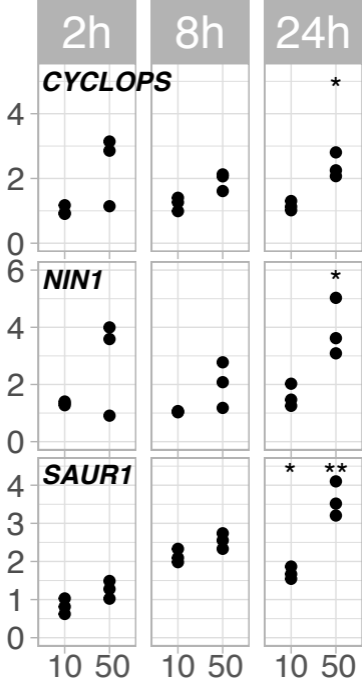

Concentration of PAA (nM)

Supplement: S4 Fig — Transcript abundance was analysed by RT-qPCR after 2h, 8h, and 24h treatments with 10 and 50 nM of PAA (n = 3 for both technical and biological replicates). Abundance of target mRNA is given relative to that of PUQ and EF1-α. X-axis shows nanomolarities of PAA. Y-axis depicts fold changes compared to control roots. Welch’s pairwise t-test with Holm’s correction highlight differences at p<0.05 (*) and p<0.01 (**). Gene names are given. (PDF) [file pone.0315798.s004.pdf]
