# Supplementary material for: The auxin phenylacetic acid induces NIN expression in the actinorhizal plant Datisca glomerata, whereas cytokinin acts antagonistically
Source: PLoS One. 2025 Feb 3;20(2):e0315798. doi: 10.1371/journal.pone.0315798 (PMC11790169; doi:10.1371/journal.pone.0315798)
Supplement: S2 Fig — Full alignment of CYCLOPS from 8 nodulating species and 1 non-nodulator (Trema tomentosa) (A). For Datisca glomerata, regions spanning disorder predicted (purple) and coil (light green) domains are depicted by a horizontal top bar. Note the presence of a disorder predicted domain at the N-terminus of Datisca glomerata that is only conserved in Ceanothus thyrsiflorus and Alnus glutinosa. Maximum-likelihood phylogenetic reconstruction of CYCLOPS/IPD3 (B). Multiple protein sequence alignment was produced after searching the orthologous group ENOG410IGIK at UniProtKB. Model legume orthologs are highlighted in pink, Ceanothus thyrsiflorus in green (GenBank accession MN388817), and Datisca glomerata in blue. (PDF) [file pone.0315798.s002.pdf]

A

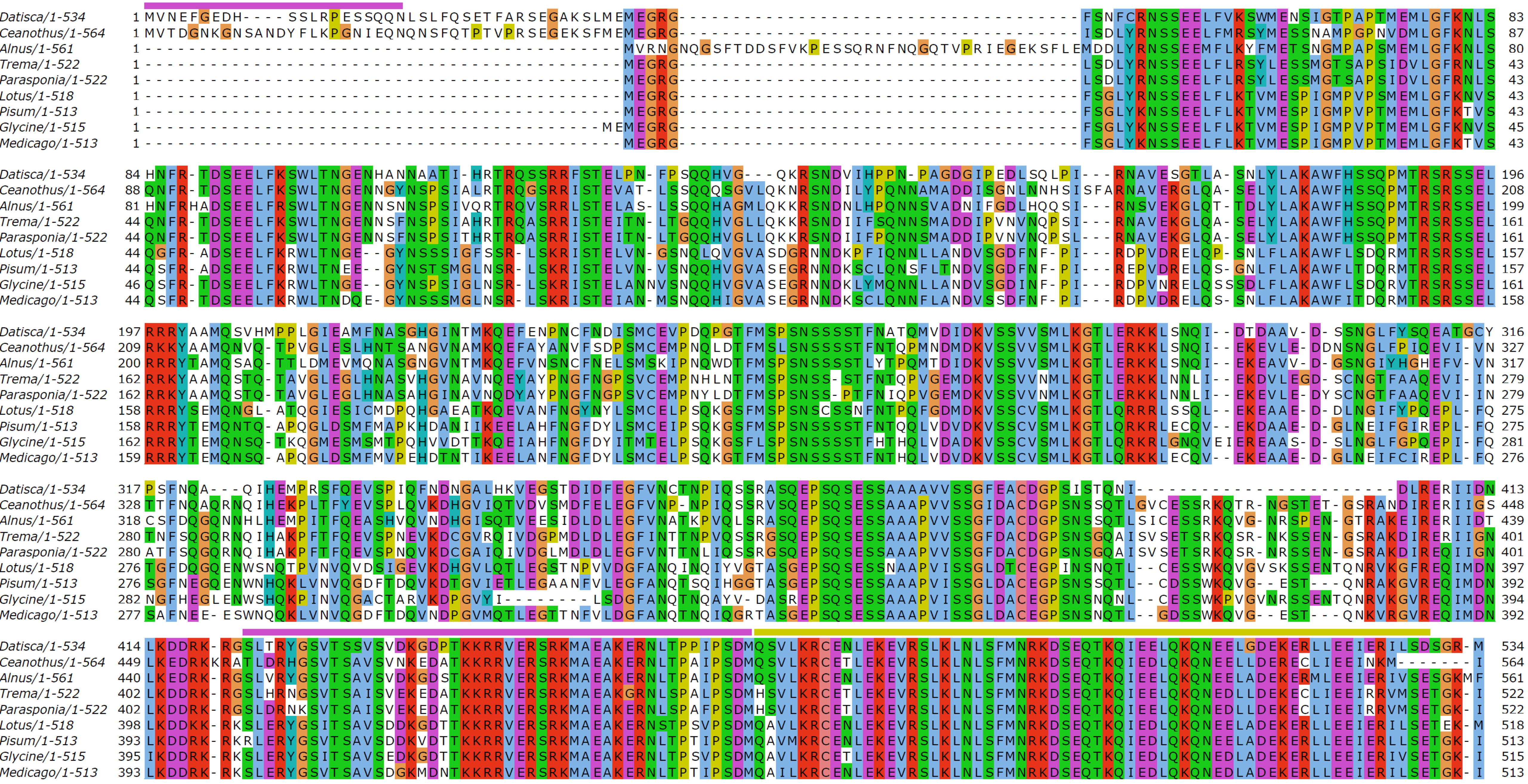

Datisca/1-534
Ceanothus/1-564
Alnus/1-561
Trema/1-522
Parasponia/1-522
Lotus/1-518
Pisum/1-513
Glycine/1-515
Medicago/1-513

B

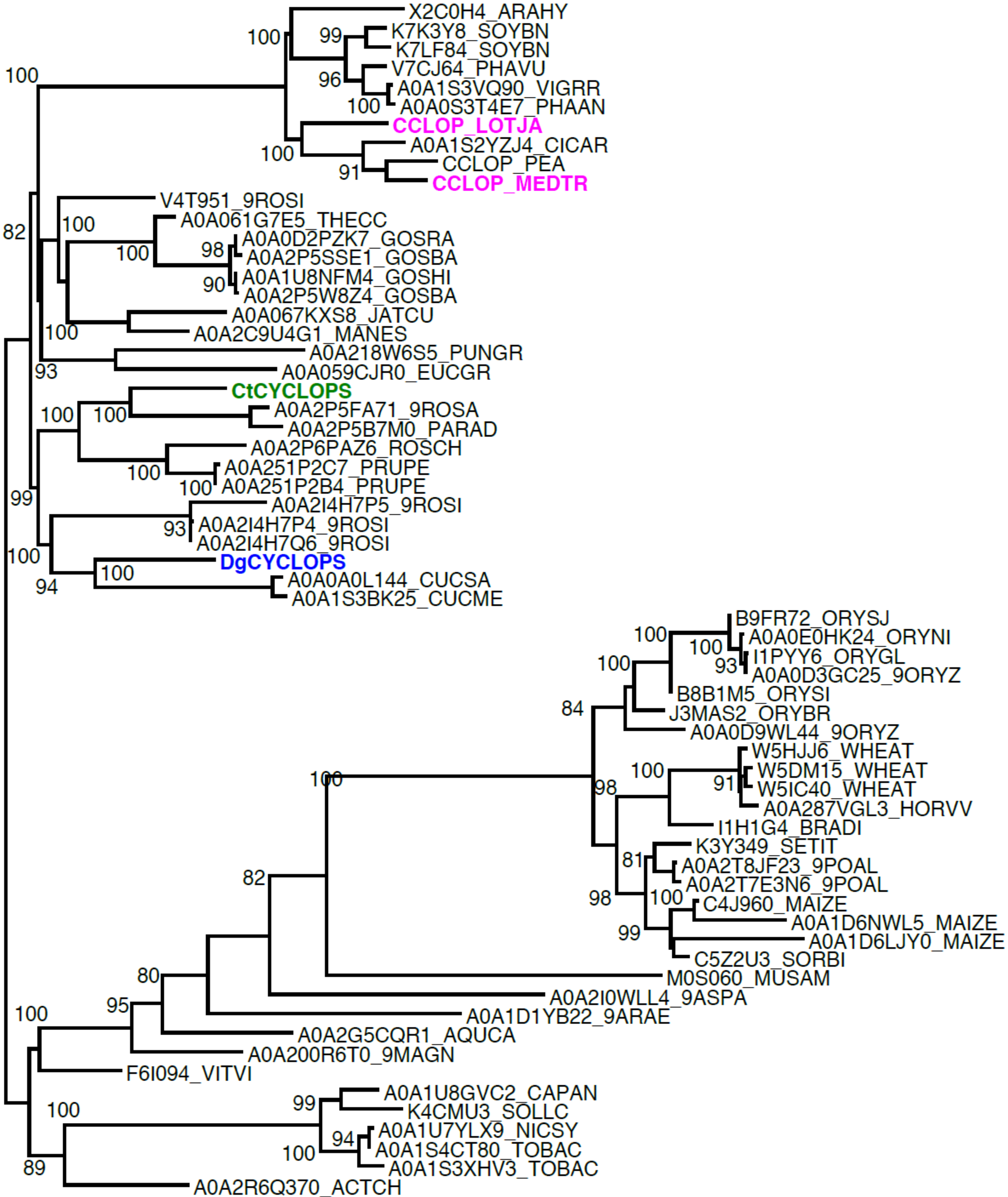
X2C0H4_ARAHY
K7K3Y8_SOYBN
K7LF84_SOYBN
V7CJ64_PHAVU
A0A1S3VQ90_VIGRR
A0A0S3T4E7_PHAAN
CCLOP_LOTJA
A0A1S2YZJ4_CICAR
CCLOP_PEA
CCLOP_MEDTR
V4T951_9ROSI
A0A061G7E5_THECC
A0A0D2PZK7_GOSRA
A0A2P5SSE1_GOSBA
A0A1U8NFM4_GOSHI
A0A2P5W8Z4_GOSBA
A0A067KXS8_JATCU
A0A2C9U4G1_MANES
A0A218W6S5_PUNGR
A0A059CJR0_EUCGR
CtCYCLOPS
A0A2P5FA71_9ROSA
A0A2P5B7M0_PARAD
A0A2P6PAZ6_ROSCH
A0A251P2C7_PRUPE
A0A251P2B4_PRUPE
A0A2I4H7P5_9ROSI
A0A2I4H7P4_9ROSI
A0A2I4H7Q6_9ROSI
DgCYCLOPS
A0A0A0L144_CUCSA
A0A1S3BK25_CUCME
B9FR72_ORYSJ
A0A0E0HK24_ORYNI
I1PYY6_ORYGL
A0A0D3GC25_9ORYZ
B8B1M5_ORYSI
J3MAS2_ORYBR
A0A0D9WL44_9ORYZ
W5HJJ6_WHEAT
W5DM15_WHEAT
W5IC40_WHEAT
A0A287VGL3_HORVV
I1H1G4_BRADI
K3Y349_SETIT
A0A2T8JF23_9POAL
A0A2T7E3N6_9POAL
C4J960_MAIZE
A0A1D6NWL5_MAIZE
A0A1D6LJY0_MAIZE
C5Z2U3_SORBI
M0S060_MUSAM
A0A2I0WLL4_9ASPA
A0A1D1YB22_9ARAE
A0A2G5CQR1_AQUCA
A0A200R6T0_9MAGN
F6I094_VITVI
A0A1U8GVC2_CAPAN
K4CMU3_SOLLC
A0A1U7YLX9_NICSY
A0A1S4CT80_TOBAC
A0A1S3XHV3_TOBAC
A0A2R6Q370_ACTCH
