# Supplementary material for: The auxin phenylacetic acid induces NIN expression in the actinorhizal plant Datisca glomerata, whereas cytokinin acts antagonistically
Source: PLoS One. 2025 Feb 3;20(2):e0315798. doi: 10.1371/journal.pone.0315798 (PMC11790169; doi:10.1371/journal.pone.0315798)
Supplement: S5 Fig — (PDF) [file pone.0315798.s005.pdf]

Supplementary Figure S5 | Shown are *cis*-regulatory elements found 2000 bases upstream of (a) *DgCYCLOPS* and (b) *DgSAUR1* coding regions, known to be involved in auxin (Cherenkov et al., 2018; highlighted in magenta) and cytokinin (Liu et al., 2019; Rashotte et al., 2003; Xie et al., 2018; highlighted in yellow) responses. Possible regions for initiation of transcription (TATA box) are highlighted in green.

A)

>*DgCYCLOPS*_promoter

ACTCTCCTCTCTCTCCTCACCTACTGTTTTTTTTAACTTTTAATCAATAAGCACGATGGTCAAATTAAAA
CATAAACTAAAGTTAAAGGGAGAGATAGAGAATTGTTATACTGTGATGATAAAATTGAAACTTAAGG
CCAACTTTGATGACTAAAATGATATTTTCCATAAGTTGCAACAGATTCAACCAAATGAGAAAGAAGA
ATAAAACGAGTACAAAAAGCATATTCTAAGAGCTAAAGAAAGTGGCACATTTTGACAACCGGCCTAA
AATCATAAGGGATAGTCTTTATTTAATGATAGGAATCGAAAGTCAAGACTGCAAATGATTAAGAATA
ATATTATAATTTACTTAAACATCATTTGCTAATTACTTATGTATGTTTTAATCCTCCATTTCAAGTTTAA
GGCATTTGAAATATATAAGTGTGATATCACATCTTTTTCAATTAAAAAAACAATTTAAGTATTAACTAT
TAAGTGGTGCAACCACGAAACTTAATAGAAACAAAAAAAAAATGCAAATATTATCATAGTCTTGTAA
TCCTTTCAGTGCCGGATTTGTCTTCCTAAACAATGCTAATAGAAAGTAACTAATCATATACTGTTAGTT
AATTAATAGCCCCTACATTTTTTCTTCAAAACTTTGATAGTATAATTTTCAGTTCATCTTTTTTTTTTTTA
AGTTGCCAAATGATTAACTTTGGTTAGAGAGCAACACAAAGAGCTATGCTAGGAGTAAATCAAAAAC
CATTAAAGTTAGCTACTTACTACAAGAAGATAAATGCATTTTCAGTTGCATATGCATAACACAAAGAG
CTATTGTTTCCTTATACATAAGTTGTTTAGATTCTCATAAGGTCTTGTTTTATGGTGTGTGGATCTGAA
ACTGTGTATACATATATAGTTTTTATGTTAGTAGTATCATGATCTCATAGAAAATGTTTTTCCATATTG
TTGTTTGCTGAAATTAACAAAGAAAATGAAAAAGAAAAGAGAGAACCAACTGTAGAGCTATTGTTGC
AAAACCCTCTTTTTGAAGCATAAAAGGGCCAAATATATAAAAGTAGACAATAAACTGTTATGAGATC
ATGAAAGCTCCATCCCAACTGCTACCATTTTTTTAAACACTGTACAGTGAGACAATTGCTGAAGGTCC
TTTTCATGGACTCATGGTTGCGTCGTTCCTTGGCTCTCCCTCGGAAGAAACCTCTATTTCTCCATCATTT
TATATCATTTCTCTGTTTTTTTTAATTCATTTATCTTACTCTGCTTATTTGTTCGAAAAGTTGAAGCTCAA
CCAAGGTATTGTGGAGGTAAATTTGACTTCTAGACCTCTAAAGCAACTGACTACGCCTAATATCTAAG
AAAAAGACAAAAACTTGGCAAATGGAATCTTGAAAAAGCCCCATAACTATTAGATTCTATGGTTTTTT
TTCCTCTGTTGTGCCCACCATTTGTTTGTGGGATTATTCAAGTGAAAGAGCACGTTAGTTTGCAGTGT
AGTCTAAAAAAATAGGGGGATTTTCATTTGACGTTTATTCCTGGATAGGTTTGAAATATGGATGTGCC
CAGCTGGTTTTATTAATGCTTGGACGGACTTTGGGAGTACAACACCTTGGAGTCAATCTTTTTGGTTG
ATTGGAATCAGAATCATGGTTTTCTTTCCTCTCTAGAAGCATATTTAAATATATATAGTTGAGGCGTA
AATTGTTGGAGAAAGTTATTCCTTTTTTCATTTTGGTGTTGAGATTTTTTAATATATGATTGATCATTTA
GTTTAATCTCCCAAAAAGGAAACTTAAATCCTAGTGGAGGTCAAAGGGTATCTGGCTATTCATCTGA
ATAGAAAGTTCAAGGATTGGGATGACATTTCAGCTTCGTTTTTGTTTGACAAGAGTGGAATAATTCAT
ATGGCGGTCTGGGTAAAATTCAAGCATATATGGTTATCCATCCTCTAGGTGTCTTCTCTTGGTTTCCCA
GATTTGAGCTAAACTAATA

B)

>*DgSAUR1*_promoter

ATGTAAATGAGGTACAAATGTACCTCCCCAGTACGGCAGAAGGACTCTGATATGATATTCAAGTTGG
ATTCCACAATTGTTATTAAAGCAAATTGGTAGACTTGCATGATTAATGTACTTAATTTAATGGACAAA
GATAAGCTAAGTTGAAGACGAGTATTTTACCCATAATACTGAGGGCCGAGGGGAAAAGGTTGGCCC
TTGGTGGGTTTGAACCATCAGGTGGAAGAGGTAATTGGGGAGATGTGGCAAATAACCCAGAAGAA
AGCAACAAAAGAAAAAGCTTATTATTTGAAATTAATATTTCAAACACAGCAAGTTGGGATAAAAAGA
GAGGAACTAATAAACGAAAAAAAACTGATAGGCATGAGAGTGAAGAATGAGTCAGAGAAATGGGA
GTGCTGTTTTATTTTATTTTTATTCTAAAAAGAACAATTGAGTACTAGTAAAGTTTTTTTTATTCAATTT
AAAATTTTTATTGGTAATTAAAGATTAAAAAATTTAGCACCTCTGTCATCGATACTTAAGTTTTAAGAA
GTACAACAGGTTGTGTTTTTTTCGAAGCATAAAGAATGAGAAATTAATTGCATTACATGCCTCAACAC
ATAAAAACATTAGCATTTATCTCAATTTCTTCAATATTTTGTTACCCCTCCCTTCTTTTTTCTAGGATTTG
ATTAATTTAATGTACATCTCAATTAATTTCACGAGACTCATGTACCGTGCACTTCTTTTTTCTAGGATTG
AGAATGTTCAATAACATCCAAAATTAGTATCAAAGTCAGATCCATATTTATTTAACAAATCTCCAGTCC
AATTCATGTGAAGTGAGTCGTTGGTCTTCCTTTGTTCTAAGATGGATGATACCCCTTGATACCAGATT
CAAAGGATTAGCTCACCGTTCGAGGCAACATAACAACATTCCTTTGGAAACCAAAAAGAATAATTTA
TTCGAGTGGAAAGTATGGTGAATCTGGGTGAAGCGACTCGTTGGCTCCCTGACTCACGGTCCGATGT
ATGGTGATTTGGGTGAAGCGAGTCGACGGGTATCCGACTTAGGCCCGACGGTTGGTAAATCCGGAT
GAAGCGAGTCGATGGTCATCCAACTCAGAACTTGATGTATGACCGGAGAGATTGCTGGAGTTATCCC
ACATCGATTGTAGAAGAGATTGATAGACAGTTTATAATTGTGAGAGAAAGACTCACCTCTTAAGCTA
GATTTTAGGGTTGAGAAAGTCCAATAACACTCAAAAGATTTTACATTAAAGAACATTTGAAACTAATA
CTTTTTATGTGGACAATAAATATTCATGAGAAAGTTAAGGGTGTTCACGGTTCAGTTCGATATTATTA
ACTAAAAATAATTTTAAATTTATTATTAAAAAAATATAAAATTAAAGTTTTATTTCAGTTCGGTTCATTT
TTATAAAAAATTAAATATAAAAATAAAAAAAATAATAAATTTAATTTTTCAATTCGGCTCGAAAACTATT
AAAATTTTAAAACGAACCGAAATTTCGATTCTATCATTTTTCTCAACCGAATGCATATAAAGTATTGAT
TAAATCGGTCATGTTTTATTTTAAACACCATAAAGAAAGTTAATGGGGTAAAGTAATTCTTGGAAATT
GAGAGGATGCTTCAGACCTAAACATCAAATAGAGACAAAGCCATTGCTTAAAAGCTCAAACCAATAG
CCAAATGTCCTTTTAGATGAGAGGGACCAAGATGTCCTTTGGAAGGACAACAATAGGAAAGAAACC
CAAAGGTATTGCCTCCAAACATGTCCTTTGGAGAACTCTCACAAACCCCAAAAAAACAACTAATGTTT
ATTAAATTTTTTTTTTTGCCCCCACAACTCCACATGACTTTGTTTCTCATTTTCACCCATAAATACCCCTA
CCTTCCTCCCCCACCCTCCTCACCTTTTACCATCTACCCCATTTTCTCTCTCTATCTCTCACAACTACTTTT
TTTCTCTCATCTAACCTCCATAACCAAA

Cherenkov, P., Novikova, D., Omelyanchuk, N., Levitsky ,V., Grosse, I., Weijers, D., and Mironova, V. (2018) Diversity of cis-regulatory elements associated with auxin response in *Arabidopsis thaliana. J. Exp. Bot.* 69, 329-339.

Liu, J., Rutten, L., Limpens, E., van der Molen, T., van Velzen, R., Chen, R., Chen, Y., Geurts, R., Kohlen, W., Kulikova, O., and Bisseling, T. (2019). A remote *cis*-regulatory region is required for *nin* expression in the pericycle to initiate nodule primordium formation in *Medicago truncatula. Plant Cell* 31, 68-83.

Rashotte, A. M., Carson, S. D. B., To, J. P. C., and Kieber, J. J. (2003). Expression Profiling of Cytokinin Action in Arabidopsis . *Plant Physiol.* 132, 1998–2011. doi: 10.1104/pp.103.021436.

Xie, M., Chen, H., Huang, L., O'Neil, R. C., Shokhirev, M. N., and Ecker, J. R. (2018). A B-ARR-mediated cytokinin transcriptional network directs hormone cross-regulation and shoot development. *Nat. Commun.* 9, 1604. doi: 10.1038/s41467-018-03921-6.
