# Supplementary material for: The auxin phenylacetic acid induces NIN expression in the actinorhizal plant Datisca glomerata, whereas cytokinin acts antagonistically
Source: PLoS One. 2025 Feb 3;20(2):e0315798. doi: 10.1371/journal.pone.0315798 (PMC11790169; doi:10.1371/journal.pone.0315798)
Supplement: Attachment — Submitted filename: Responses_to_reviewers_comments.docx [file pone.0315798.s008.docx]

**Responses to reviewers’ comments**

Authors:

First, we would like to thank all reviewers for their efforts. You will see that we had to rewrite the manuscript to some extent – originally, these data were obtained in 2017-2019, and we did not check the promoters of the two *NIN* genes of *D. glomerata* in the beginning of the project. The fact that the *DgNIN1* promoter contains a non-standard PACE element – which nevertheless works as PACE element, see Cathebras et al. (2022) – has prompted us to include some bioinformatic analysis on both *PACE* elements from *D. glomerata* and DgCYCLOPS (added to Fig 2 and S2 Fig).

Reviewer #1: In comparison with legumes, genes invovled in actinorhizal nodule organogenesis remain less understood. In this study, the authors analyzed the responses of several nodulation genes in Datisca glomerata hydroponic system.This study supports the view that cytokinin signaling is central for cortex-induced nodules of L. japonicus but acts as an antagonist to the induction of pericycle-induced nodules of D. glomerata by PAA The results would be potentially interesting to the field and teh findings are interesting. I have several specific comments:

Reviewer #1-1:

The authors showed candidate gene expression in response to phytohormone treatment . However, no data was presented how the phytohormones affect nodule number or formation. Physiological observation should be performed regarding the nodulation phenotype in different treatments.

Response to #1-1:

This would problematic for the following reasons:

- Because the microsymbiont of *D. glomerata*, *Candidatus* *Frankia datiscae Dg1*, cannot be cultured, (a) we have no way to quantify the inoculum, and (b) we have to infect with crushed nodules of older plants, which means we have a non-sterile inoculum. We have to work in the greenhouse.
- *Datisca* seeds are similar in size to *Arabidopsis* seeds, not as large as the nutrient-rich seeds of legumes. Seedlings do not form nodules. Plants have to be several weeks old (at least 7 cm high) when they are predisposed to be nodulated.
- Nodulation of actinorhizal plants is not as fast as for legumes. We first find nodules *ca.* 6 weeks after infection. This timing is for Sweden in summer (nodulation in California is faster). Nodulation in Sweden in Winter is slower (8-10 weeks).

Thus, we would be dealing with plants in 12 cm diameter pots (*Datisca* root systems are large). How are we to add the phytohormones – every three days, once per week? What role does the soil microbiome play? How does the phytohormone distribute in the pot? We tried this once before [for Demina et al. (2019) with water *vs.* NAA; NAA is at least known to be stable] and the standard deviations were enormous; we ended up not including the results in the manuscript.

Reviewer #1-2:

The picture of the hydroponic culture should be moved from supplemental figure to Fig.1.

Response to #1-2:

Fig S1 has been moved to Fig 1, and the other Figures have been renumbered.

3. Phylogenetic trees should be shown for the genes used in this study, demonstrating that these are indeed orthologs of nodulation genes in legumes.

Response to #1-3:

These trees are already included in the supplement (Supplementary Figures S2-S4 in the previous version, Supplementary Figures S1-S3 in the revised version).

4.The agricultural significance of legume and actinorhizal nodulation should be introduced in the first paragraph. For example, nodulation in legumes reduce fertilizer usage and improves soybean yield (<https://doi.org/10.1038/s41477-024-01696-x> ).

Response to #1-4:

This has been done: “While legume symbioses are essential in agriculture insofar as they yield protein-rich seeds while rendering their host plants with an independent source of nitrogen fertilizer, thereby contributing to increases of nitrogen pools in surrounding ecosystems, actinorhizal species mostly represent pioneer plants and are often used in reforestation or soil reclamation [Andrews et al. 2011].”.

**Reviewer #2**: In the manuscript Salgado et al., describes the primary hormonal response in primary symbiotic specific transcription factors after treatment with phytohormone. The major take home massage of this paper is that in the actinorhizal plant Datisca glomerata the gene expression of NIN induces after auxin (PAA) treatment instead of CK (BAP) treatment. This is an important massage for the nodule biologist. Despite this fact, there are some important lacunae in the manuscript that need to be addressed.

Major points:

Reviewer #2-1:
NIN1 expresses in root and eventually induces in nodule where as NIN2 is nodule specific. Further, In the figure 1 where both NIN1 and NIN2 expression has been shown there it is clear that NIN2 expression is higher than NIN1. If so, then why the complete study is conducted only using NIN1. Only the proximal promoter analysis can not be used as an logic to not conduct the qRT-PCR analysis of NIN2.

Response to #2-1:

As pointed out, *NIN2* is expressed in a nodule-specific manner, while the experiments for this manuscript were performed on roots. We could not induce nodules in an axenic system since the microsymbiont of *D. glomerata*, either *Candidatus Frankia datiscae* or *Candidatus Frankia californiensis*, is not cultivable. We could not detect *NIN2* levels of expression on either roots of greenhouse-grown plants or on roots from our hydroponic system, independent of the presence of exogenous phytohormones. We cannot induce nodules in the hydroponic system, because we do not have axenic inoculum (see Response to #1-1). (Nodulation is possible under hydroponic conditions provided the cultures are aerated, but this requires large cultures started with 6-weeks-old plant, and there is significant growth of non-Frankia bacteria).

Thus, while the promoter of *NIN2* contains the PACE element (Cathebras et al. 2022) and thus is induced by the CCaMK/CYCLOPS complex in response to bacterial signalling, it was impossible to examine its induction using plantlets growing in an axenic hydroponic system.

Cathebras et al. (2022) have shown that the *DgNIN2* promoter contains a standard *PACE* element while the *DgNIN1* promoter harbors a non-conventional *PACE* element (see Fig 2B,C). Nevertheless, experiments of Cathebras et al. (2022) using *D. glomerata* *PACE* elements driving the expression of *NIN* were able to complement and rescue the *Lotus japonicus* nin15 phenotype. We have added a large paragraph in the Result section of the manuscript about *cis*-regulatory *PACE* elements in *D. glomerata* as well as about their presumable target, DgCYCLOPS. This section was included to get a better understanding on whether *PACE*-CYCLOPS events in *D. glomerata* are supported from an *in silico* perspective. It reads now:

“*D. glomerata* contains two copies of *NIN* [3] and although both *NIN1* and *NIN2* are induced in nodules compared to roots, only *NIN2* is expressed nodule-specifically with no detectable levels of expression in roots of either seedlings or greenhouse-grown plants (**Fig 2;** for *NIN1* see also [55]). Cathebras et al [54] have shown the presence of the *cis*-regulatory element *PACE* within the promoter of *DgNIN2* (termed *DgNIN1* in [54]) as a requirement for induction by the CCaMK/CYCLOPS complex, whilst the promoter of *DgNIN1* contains an uncommon and extended version of *PACE*, which compared to that of *DgNIN2*, or those of other root nodule-forming plants, contains an insertion of eight nucleotides at the core of *PACE* and displays high nucleotide conservation across *PACE-Y* and *PACE-X,* and *ca.* 67% conservation at the core of the *PACE* element (**Fig 2B)**. To gain a better understanding of the *PACE*-CYCLOPS interactions in *D. glomerata,* we compared the amino acid sequence of DgCYCLOPS with those of CYCLOPS proteins from a range of root nodule-forming plants and one non-nodulator, *Trema tomentosa*. Alignment of CYCLOPS orthologs showed that the primary structure of CYCLOPS, along with its ortholog in *Ceanothus thyrsiflorus*, displays a ~40 amino acid sequence extension at its N-terminus that is neither present in *Alnus glutinosa*, *Parasponia andersonni*, nor in the legumes included in the analysis (**S2A Fig**); importantly, the first 20 amino acids represent a disordered domain whose presence at the N-terminus seems to be a feature of actinorhizal plants as it was only found in *Datisca glomerata* (position 1-20), *Alnus glutinosa* (position: 1-33) and *Ceanothus thyrsiflorus* (position 16­-36) (**S2A Fig**). Furthermore, 17 amino acids upstream of the largest and well-conserved disordered domain, placed at the C-terminus, the primary structure of DgCYCLOPS shows high dissimilarity with that of other orthologs (**Fig 2D**; full alignment in **S2A Fig**). To investigate whether this dissimilarity could lead to alterations in protein secondary structure, AlphaFold structure predictions were carried out and the comparison of the predicted models showed the presence of an *α*-helix in *Datisca glomerata* CYCLOPS that is absent in CYCLOPS proteins of *Ceanothus thyrsiflorus*, *L. japonicus*, and *Medicago truncatula* (**Fig 2E**).”

Minor points.

Reviewer #2-2:
1. In the page 3 in introduction the involvement of the phytohormone para written in an confusing manor.

Response to #2-2:

We have rewritten the entire part that deals with phytohormones.

Reviewer #2-3:
2. Page 4, ‘suggesting that DgNIN1 signaling might dependent on auxin or cytokinin’. There should be auxin and/or cytokinin.

Response to #2-3:

We apologize for the mistake. This has been fixed now.

Reviewer #2-4:

3. Page5. Preliminary assays confirmed ARR9 as a suitable marker gene for cytokinin in D. glomerata, owing to an increase of ARR9 mRNA levels in the presence of BAP (discussed below), please add figure number here.

Response to #2-4:

This has been fixed: “Preliminary assays confirmed ARR9 as a suitable marker gene for cytokinin in *D. glomerata,* owing to an increase of ARR9 mRNA levels in the presence of BAP (see below in Fig. 4).”

Reviewer #2-5:

4. In Fig 1, the statistics is not marked as mentioned in the legend. For all the figure please mention the number of technical and biological replicates.

Response to #2-5:

We apologize for the omission. This has been fixed now.

Reviewer #2-6:

5. The transcriptional profile exhibited by CYCLOPS in this assay indicates that its transcription is tightly regulated by BAP levels within the range of 10 nM to 100 nM. Why the CRE1 cytokinin receptor gene was not taken into study? I suggest to check CRE1 expression as well.

Response to #2-6:

Our interest was focussed on the common symbiotic signalling pathway that leads to Calcium spiking, activation of CCaMK, activation of CYCLOPS and finally, activation of NIN. We included one of the next genes in the cascade, *NF-YA1*, to confirm *NIN* transcription. We discussed the inclusion of other genes when we had the first PAA results. CRE1 was eliminated early in the game because (a) we not know whether CRE1 is involved in the nodulation program of actinorhizal plants in general and *Datisca glomerata* in particular (see van Zeijl et al. – it is not even clear whether it is involved in nodulation in *Parasponia*), and we had/have neither the time nor the (wo)manpower to perform CRISPR/Cas9 hairy root experiments, (b) the induction of *CRE1* transcription by exogenously applied cytokinin in *Medicago truncatula* is rather weak. Furthermore, our results in this study do not point at cytokinin accumulation as a factor in nodule organogenesis in *D. glomerata.* At any rate, the main objective was to find a reliable molecular readout for BAP, and *ARR9* fitted that need pretty well as it behaved in a rather consistent manner throughout our experiments.

Reviewer #2-7:

6. The expression of L. japonicus cytokinin oxidase/dehydrogenase3 (LjCkx3; [56]) was induced by BAP when applied for a period of 8h. In the Fig 2, if auxin and BAP are antagonists then why CKX3 expression did not decrease in PAA, NAA treatment? Please elaborate.

Response to #2-7:

Fig 3 (Fig 2 in the previous version) does not show the effects of combinations of phytohormones. According to our results, BAP acts as antagonist of PAA in *D. glomerata*. It should not act as an antagonist of NAA or PAA in *L. japonicus* since here, all three phytohormones induce NIN expression when applied individually.

Reviewer #2-8:

7. D. glomerata roots exhibits a different temporal window to react to possible effects of cytokinin when compared with roots of L. japonicus, which could be best explained by differences in root development between the two species. Please elaborate few lines about root developmental difference between these two species.

Response to #2-8:

We have inserted this text:

“The different temporal window could be explained by the fact that while legume seeds are large and nutrient-rich [Mathesius 2022] which means that legume seedlings can afford the carbon expense required for nodule formation, seeds of *D. glomerata* are similar in size to those of Arabidopsis, and greenhouse plantlets do not nodulate before they have reached a height of at least 5 cm (K. Pawlowski, unpublished observations).”

Reviewer #2-9:

8. PBZ-treated roots showed a significant decrease in mRNA abundance of CYCLOPS and NF-YA1 when compared with their control and AVG-treated counterparts. The significance was not marked in the Fig 5.

Response to #2-9:

The reduction of expression levels as a consequence of PBZ treatment is significant for CYCLOPS, but not for NF-YA1. This has been corrected in Fig 6 (Fig 5 in the previous version).

Discussion
Reviewer #2-10

9. Since the induction of the actinorhizal nodule primordium takes place in the root pericycle [42], the same mechanism would not necessarily be expected for actinorhizal plants. Please elaborate this and connect with the provided evidences from this study.

Response to #2-10:

The sentence has been replaced with: “Legume nodule primordia are induced in the root cortex and the root pericycle, while actinorhizal nodule primordia are induced in the root pericycle. Therefore, we do not necessarily expect the same involvement of phytohormones in both processes.”

Reviewer #2-11:

10. Indeed, the results of this study showed unambiguously that the expression of D. glomerata NIN1 is repressed by the synthetic cytokinin BAP and induced by the natural auxin PAA, though not by the synthetic auxin NAA. How the LjNIN and DgNIN1 is receiving this application of exogenous PAA and NAA in different style? Can you add some comments about its signalling?

Response to #2-11:

We would like to stress that the antagonism between BAP and PAA works not only for the induction of *DgNIN1*, but also for the induction of *DgSAUR1*. However, since NAA and PAA were shown to be transported differently in Arabidopsis (Sugawara et al. 2015), we do not expect that a PAA antagonist will necessarily work as an NAA antagonist. PAA is the dominant auxin in *D. glomerata* (Demina et al. 2019); its distribution in other actinorhizal plants has yet to be examined. PAA could not be detected in *Lotus japonicus* at all (Ng and Mathesius 2022), so we feel that for the examination of PAA effects, *L. japonicus* is not the ideal model system.

Reviewer #3: Comments
The study conducted by Salgado et al, focused on the genes and phytohormones requirements during the nodulation program of the actinorhizal plant Datisca glomerata, a largely unknown process. The purpose of the study is clear and very well written. This working model represents numerous challenges, that limit the approaches employed in model legumes. Nonetheless, they obtained interesting results on the differential responses of D. glomerata roots on symbiotic gene expression, after treatment with synthetic phytohormones, specially cytokinin. Undoubtedly this work provides valuable information to the community interested in plant-microbes mutualistic associations.

Response: We thank the reviewer for these positive remarks!

Minor comments
Reviewer #3-1:

Strictly talking, the title of the manuscript is correct, but probably not very “catchy”. The authors could consider a title that includes the terms hormones and remove Phenylacetic acid. The different phytohormone-related gene expression might be highlighted.

Response to #3-1:

We would rather keep the reference to PAA in the title because we feel that the role of PAA in the regulation of *NIN* is one of our most important results. PAA is an under-researched auxin; the role of PAA in legume nodule formation has not been examined yet. We have changed the title to “The auxin phenylacetic acid induces *NIN* expression in the actinorhizal plant *Datisca glomerata*, whereas cytokinin acts antagonistically*”.*

Reviewer #3-2:

Page 1. “In all these interactions, the microsymbionts are not vertically transmitted”. In common bean and probably other legumes, rhizobia can be vertically transmitted (<https://doi.org/10.1111/j.1574-6941.1998.tb00513.x>)

Response to #3-2:

We have changed this sentence: “In all these interactions, the microsymbionts are not vertically transmitted; although the microsymbionts can exceptionally be carried by host-produced seeds [7], nodule induction always starts with bacteria in the soil, not with bacteria in the plant.”

Reviewer #3-3:

Results: The length of the subsections can be shortened
Figure Legends: Please include the number of harvested plants per biological replicate.
Cytokinin and cytokinin-related genes seem to be differentially required in “unusual” nodulation programs, for instance the nodulation in Parasponia (doi: 10.3389/fpls.2018.00284) and the intercellular infection program in Lotus japonicus (<https://doi.org/10.1093/plphys/kiaa049>). The authors could discuss more about it.

Response to #3-3:

We would rather not shorten the subsections in Results. We are aware that some sentences could be moved to the discussion, but that would make the whole thing less accessible to the reader; it is easier to assimilate the information when having the Figures in view.

Regarding the unusual nodulation programs, we have added this: “It has to be pointed out that in the only non-legume nodulated by rhizobia, *Parasponia* spp., the involvement of cytokinin signalling in nodule organogenesis is not yet clear since knockout mutants of one cytokinin receptor are not affected in nodulation; this may be a result of receptor redundancy – or not [van Zeijl et al.]. The role played by cytokinin in nodulation was also changed when the intercellular infection pathway of the model legume *Lotus japonicus* was examined, in that knockout mutants of the cytokinin receptor showed a much stronger phenotype than those used for intracellular infection studies [Montiel et al.].”

Reviewer #4: The present study focuses on hormonal regulation of genes encoding key regulators of symbiotic nodules development in actinorhizal plant Datisca glomerata. Molecular mechanisms underlying the symbiotic nodule development and its hormonal control is well established for legume-rhizobia symbiosis; however, for actinorhizal plants, its regulation remains less understood. This study is aimed to fill this gap. Overall, the results of this study are interesting and propose that key regulators of symbiosis are regulated differently in legumes and actinorhizal plant D. glomerata: notably, the expression of the DgNIN gene is induced by auxin (phenylacetic acid), but not by cytokinin BAP as it was shown for L. japonicus and other model legumes. However, the obtained results require further clarification and discussion.

Reviewer #4-1:
1. The inhibitory effect of BAP on expression levels of CYCLOPS, NIN1 and NF-YA1 was shown for 30-day-old plants. However, at this age, in a trial experiment using roots of 30-day-old D. glomerata seedlings, the exogenous application of 50 nM PAA did not lead to any changes in expression of either CYCLOPS or NIN1 in 24h treatments. Therefore, authors suggested the possibility that root development could have a pivotal influence on the transcription of CYCLOPS and NIN1 at this stage (30-day-old D. glomerata seedlings). In 54-day-old seedlings, 10 and 50 nM PAA induced NIN1 expression, however, in this experiment, 10 and 50 nM BAP did not downregulate NIN1 activity at this stage (10 nM BAP even tends (although non-significantly) to increase NIN1 expression, Figure 4). Therefore, the conclusion made by the authors (throughout the Discussion section) that BAP inhibits NIN expression in D. glomerata is not quite correct. These findings should be discussed more accurately. The effect of BAP and auxin treatment on gene expression levels in D. glomerata should be compared at the same developmental stage.

Response to #4-1:

We have explained the effect of the state of root development by sink/source regulation – *D. glomerata* seedlings have to have reached a certain age before they can nodulate. We write now in Results: “The different temporal window could be explained by the fact that while legume seeds are large and nutrient-rich [Mathesius 2022] which means that legume seedlings can afford the carbon expense required for nodule formation, seeds of *D. glomerata* are similar in size to those of Arabidopsis, and greenhouse plantlets do not nodulate before they have reached a height of at least 5 cm (K. Pawlowski, unpublished observations).”

and in the Discussion in more detail: “The difference between the reaction of 30-day-old *D. glomerata* seedlings to phytohormones compared to 52- or 54-day-old seedlings can be explained by the fact that nodulation requires a considerable carbon investment which has to be supplied from seeds or from photosynthesis. Legume seedlings are routinely nodulated in Petri dishes, *i.e*., very young, given that they obtain most of their nutrients from their large seeds. This is also possible for seedlings of the actinorhizal shrub *Coriaria myrtifolia* which forms seeds similar in size to those of *L. japonicus* (Gtari et al., 2015). However, in 30-days-old *D. glomerata* seedlings grown from tiny seeds, nodulation has to be downregulated, a process that would likely abolish or at least weaken the effects of exogenous application of phytohormones on *DgNIN1* expression, as was observed in this study – no effect of PAA was found at all, and BAP only acted at 500 nM.” Thus, the fact that BAP has a negative effect on *DgNIN1* expression even in seedlings that are too young to nodulate is significant. When talking about these seedlings, we carefully refer to BAP “abolishing the induction of *DgNIN1* by PAA”. But we would prefer not to omit the fact that a direct negative effect of BAP on *DgNIN1* expression is not unlikely.

Reviewer #4-2:
2. Expression of NIN1 is induced in nodules compared to roots, while NIN2 is expressed nodule-specifically. Since NIN1 expression is not nodule-specific, can this gene have functions in root development that are not related to the development of symbiotic nodules, but some other functions, possibly related to the development of lateral root? It would be nice to discuss this issue. Why only NIN1 expression was checked, not NIN2, which is nodule-specific?

Response to #4-2:

Regarding the first question: in legumes, *NIN* is expressed specifically in response to rhizobial Nod factors (see, e.g., https://www.frontiersin.org/journals/plant-science/articles/10.3389/fpls.2023.1284720/full) – *i.e.*, early in the nodulation process – and remains expressed in nodules. Furthermore, its expression can be induced by exogenous cytokinins. This is usually summarized as “nodule-specific expression.”

Yes, *NIN1* expression in roots could be related to more effects than nodulation – for instance, an LCO-responsive NIN gene was analysed in poplar (Irving et al. 2022; <https://pubmed.ncbi.nlm.nih.gov/35929094/>), and this role has been confirmed for the nodulation-related legume *NIN* genes. A reference has been added to the text:

“The possibility that the expression of *DgNIN1* is linked to a role in the regulation of lateral root formation, as was found for lipochitooligosaccharide Nod factor-responsive *NIN* genes of legumes and poplar [86], should be considered, however the fact that the expression of *DgNIN1* cannot be induced in plantlets too young to nodulate (**Fig 4**) and it shows high levels of expression in nodules (**Fig 2**) make it clear that *DgNIN1* has to play a relevant role in nodulation.”

Concerning the question why only *NIN1*, and not *NIN2* expression was examined, please see Response to #2-1.

Reviewer #4-3:
3. Previously, it was reported that CYCLOPS binding site and CE-region (containing many putative cytokinin response regulator binding sites) are conserved in legume NIN promoters (Liu et al., 2019; Liu and Bisseling. 2020, doi: 10.3390/genes11070777). CYCLOPS/IPD3 binding site is located about 3 kb upstream of the start codon, whereas CE-region is located far upstream from the NIN start codon (in Medicago it is about 18 kb upstream, and in Lotus it is about 45 kb upstream of the NIN start codon) (Liu et al., 2019, DOI: 10.1105/tpc.18.00478 ). It was suggested that the gain of the CYCLOPS binding site and CE-region was essential step to establish NIN expression during nodule formation.
Are the CYCLOPS-binding site and CE-region conserved in NIN1 and NIN2 genes in D. glomerata as well? It should be checked and discussed in the manuscript in order to understand the evolutionary conservatism of NIN regulation in actinorhizal plants.

Response to #4-3:
The relevance of a region with cytokinin response regulator binding sites in a distance of 3-45 kb from the *NIN* transcriptional start site to the regulation of *NIN* expression would require experimental proof. We really cannot afford hairy root experiments at this point. Furthermore, it should not be forgotten that  the B-type RR transcription factor GmRR11d is an important effector of autoregulation of nodulation and represses *GmNIN1a* expression (<https://www.nature.com/articles/s41467-022-35360-9>), explaining the negative effects of shoot-derived cytokinin on nodulation. Thus, the effects of cytokinin on legume *NIN* expression are highly complex, and without more experimental evidence, we do not see how we could meaningfully contribute to the role of cytokinin in the nodulation of actinorhizal plants. Plus, our data do not hint at any positive role of cytokinin in nodulation of *D. glomerata*.

Concerning transactivation of *NIN* by CCaMK/CYCLOPS binding, this is ascribed to the *cis*-regulatory element *PACE* (Cathebras et al. 2022). We included a large paragraph in the Results section stating that the *DgNIN2* promoter contains a standard *PACE* element (and its function was confirmed by Cathebras et al. (2022), while the *DgNIN1* promoter contains a non-standard one. Nevertheless, Cathebras et al. (2022) showed that both *PACE* elements will be induced by the CYCLOPS/CCaMK complex in *Lotus japonicus* (see response to #2-1).

Reviewer #4-4:
4. Gauthier-Coles et al., 2019 (DOI: 10.3389/fpls.2018.01901) reported that nodulating legumes are distinguished by a sensitivity to cytokinin in the root cortex leading to pseudonodule development. However, in the study by Gauthier-Coles et al, a nodulating actinorhizal species Alnus glutinosa formed no pseudonodules after application of cytokinin. In contrast, Rodriguez-Barrueco et al., 1973 (DOI: 10.1111/j.1399-3054.1973.tb03107.x) showed that cytokinin (2IP) application induced pseudonodules on A. glutinosa. Therefore, whether exogeneous cytokinin triggers cortical cells in actinorhizal species remains unclear. It would be nice to clarify this and discuss these findings in accordance with the results obtained in the present study.

Response to #4-4:

Thank you very much for this comment. We have added these sentences to the revised version:
“Our results support the observations of Gauthier-Coles et al. (2019) for *Alnus glutinosa* who showed that here, cytokinin (*cis*-zeatin, *trans*-zeatin, dihydro-zeatin, and kinetin were used) does not act as positive regulator of pseudonodule development as it does in legumes. Rodriguez-Barrueco et al. (1973), on the other hand, found pseudonodule development in response to treatment with two different cytokinins, 2-isopentenyl adenin (2IP) and kinetin; however, the latter study was not performed under axenic conditions, which means that other phytohormones produced by bacteria or fungi may have been involved.”

Reviewer #4-5:

5. The beginning of Abstract should be re-written in order to make it clearer and more consistent.

Response to #4-5:

The abstract has been rewritten.

Reviewer #4-6:

6. In conclusion, it is desirable to add a scheme illustrating the hormonal regulation of the expression levels of key nodule regulators in legumes such as L. japonicus and actinorhizal plant D. glomerata in order to summarize the findings of this study.

Response to #4-6:

A scheme has been added as Fig 7.
