# Supplementary material for: The auxin phenylacetic acid induces NIN expression in the actinorhizal plant Datisca glomerata, whereas cytokinin acts antagonistically
Source: PLoS One. 2025 Feb 3;20(2):e0315798. doi: 10.1371/journal.pone.0315798 (PMC11790169; doi:10.1371/journal.pone.0315798)
Supplement: Attachment — Submitted filename: Responses to reviewers comments_ka_3010.24_MS_final.docx [file pone.0315798.s009.docx]

**Responses to reviewers’ comments Manuscript PONE-D-24-19931R1**

**Reviewer #5 comment 1:**

After carefully reading the revised version, I came to know that the authors have successfully incorporated all the suggested changes and fully addressed the comments/changes proposed by honorable reviewers. I do not have much comments but only few suggestions that can be considered for further improvement.
It is really embarrassing to review the manuscript having no line number.
I have only few questions
1- How cytokinin and auxin interact in regulating DgNIN1 expression in Datisca glomerata?

**Response to #5-1:**

We do not know how the signal transduction pathways of auxin (here, PAA) and cytokinin interact during nodule induction on *D. glomerata*. We can only discuss our results in the context of previous studies carried out on legume mutants and the information on the nodule physiology of *D. glomerata* available to date. NIN was, based on results obtained on legumes (Soyano et al. 2015, doi: 10.1093/pcp/pcu168; Shen and Feng 2024 doi: 10.3389/fpls.2023.1284720) and the actinorhizal species *Casuarina glauca* (Clavijo et al. 2015 doi: 10.1111/nph.13506), recruited for infection and nodule organogenesis. Since in contrast with legume nodule primordia, nodule primordia of *D. glomerata* are induced in the pericycle, placed at the vicinity of protoxylem poles and where auxin levels are expected to be higher, it is not surprising that auxin, and in particular the most abundant auxin in *D. glomerata*, PAA, plays a pivotal role regarding *NIN* expression which should be required for nodule organogenesis.

We had written in the discussion:

“…the induction of legume *NIN* expression by exogenously applied cytokinin had been ascribed to the fact that cytokinin signalling takes place in the root cortex in the course of legume nodule organogenesis, leading to the formation of the legume nodule primordium [17,18]. On the other hand, shoot-derived cytokinin transported in the phloem has been implicated in the systemic repression of nodulation during the autoregulation of nodulation [78]. Legume nodule primordia are induced in the root cortex and the root pericycle, while actinorhizal nodule primordia are induced in the root pericycle [46]. Therefore, we do not necessarily expect the same involvement of phytohormones in both processes.”

We have added now:

“For induction of an organ primordium in the root pericycle, close to the auxin maximum at the protoxylem pole [de Smet et al. 2007], auxin would be expected as inducer.”

Reviewer #5 comment 2:

2- What is the role of ethylene and gibberellic acid in PAA-induced expression of DgNIN1?

Response to #5-2:

The roles of ethylene and gibberellic acid are well examined in legumes (exogenously supplied ethylene inhibits the common symbiotic signalling pathway, Oldroyd et al. 2001, doi: 10.1105/TPC.010193); for actinorhizal plants, however, we can only refer to some data on the effect of exogenous application of ethephon on the nodulation of *Casuarina glauca* (Casuarinaceae, Fagales; Ngom et al. 2020 doi: 10.1007/978-1-0716-0142-6_9) which does not belong to the same order as *D. glomerata* (Cucurbitales) and has a different infection mechanism for *Frankia* (namely via root hairs). Ethephon (which at a pH of 5 and above decomposes to form ethylene), when applied exogenously at a concentration of 50 µM, inhibits the nodulation of *C. glauca*. Thus, the negative effect of exogenously supplied ethylene on *NIN* expression in legumes as well as *C. glauca* is not present in *D. glomerata*.

We wrote in the discussion (previous version):

“In short, the effect of AVG in combination with PAA resembled the effect of the combination of BAP and PAA. This effect could be ascribed to increased amounts of cytokinins as in legumes, ethylene negatively regulates cytokinin accumulation during nodule induction [19]. Another possible explanation could involve the role of ethylene in the transport of auxin from the shoot to the nodulation site, shown to result in high auxin accumulation [34] and implying that endogenous auxin levels in AVG-treated roots could be below the level required for the induction of *DgNIN1*. At any rate, the fact that the inhibition of ethylene biosynthesis abolished the induction of *DgNIN1* expression by PAA in *D. glomerata* roots (p=0.77) was the second relevant difference found between *D. glomerata* and legumes with regard to phytohormone effects on *NIN* expression [33].”

We have now added the information on *C. glauca*:

“In short, the effect of AVG in combination with PAA resembled the effect of the combination of BAP and PAA. This effect could be ascribed to increased amounts of cytokinins as in legumes, ethylene negatively regulates cytokinin accumulation during nodule induction [19]. However, it has to be mentioned that in the actinorhizal species *Casuarina glauca,* whose nodule primordia form in the root pericycle like in *D. glomerata,* ethylene has a negative effect on nodulation like in legumes [Ngom et al. 2020]. Another possible explanation could involve the role of ethylene in the transport of auxin from the shoot to the nodulation site, shown to result in high auxin accumulation [34] and implying that endogenous auxin levels in AVG-treated roots could be below the level required for the induction of *DgNIN1*. At any rate, the fact that the inhibition of ethylene biosynthesis abolished the induction of *DgNIN1* expression by PAA in *D. glomerata* roots (p=0.77) was the second relevant difference found between *D. glomerata* and legumes with regard to phytohormone effects on *NIN* expression [33].”

No data on gibberellic acid and actinorhizal nodules are available; in legumes gibberellins inhibit infection thread formation but promote nodule organogenesis (McAdam et al. 2018, doi: 10.1093/jxb/ery046). A DELLA protein, a transcription factor that represses the reaction to gibberellin, is part of the CYCLOPS/CCaMK complex (Pimprikar et al. 2016, doi: 10.1016/j.cub.2016.01.069), a conserved module in arbuscular mycorrhization and legume nodulation, and thus expected to be conserved in actinorhizal nodulation as well, as actinorhizal plants use the common symbiotic signalling pathway (shown for *C. glauca* and *D. glomerata,* Gherbi et al. 2008, doi: 10.1073/pnas.0710618105; Markmann et al. 2008, doi: 10.1371/journal.pbio.0060068) and contain *NIN* genes (Griesmann et al. 2018, doi: 10.1126/science.aat1743) the function of which was proven for *C. glauca* (Clavijo et al. 2015, doi: 10.1111/nph.13506). When gibberellin binds its receptor, the receptor binds DELLA and causes its degradation, thereby activating gibberellin responses. In *Lotus japonicus,* gibberellin induces *NIN* expression directly via a *cis*-acting element in the *NIN* promoter (Akamatsu et al. 2021, doi: 10.1111/tpj.15128). Thus, we expected an influence of gibberellin on actinorhizal nodulation due to DELLA being part of the conserved module connecting the common symbiotic signalling pathway and *NIN* expression, but we could not predict the direction of the influence as the organogenesis of legume and actinorhizal nodules differs. It turned out that in *D. glomerata,* gibberellin was required for the induction of *NIN* expression by PAA.

We wrote in the discussion about the effects of the inhibitor of gibberellin biosynthesis, PBZ:

“At any rate, in the presence of PBZ, PAA did not cause significant changes in the expression levels of any gene examined, including that of *SAUR1* (p=0.25; **Fig 6**). Yet, in the presence of PBZ, the expression of *DgCYCLOPS* was significantly reduced (**Fig 6**), suggesting that the induction of *DgCYCLOPS* expression involves a DELLA protein. Combined, these results are consistent with the positive involvement of gibberellin signalling in legume nodule organogenesis mediated by the GRAS transcription factor DELLA1 as shown in *Medicago truncatula* [24].”

(Since a direct effect of gibberellins on *NIN* expression as shown for the *LjNIN* promoter could not be tested in our system, we did not discuss/cite Akamatsu et al. 2021.)

Reviewer #5 comment 3:

3- How do nodule formation processes in Datisca glomerata differ from those in Lotus japonicus?

Response to #5-3:

Nodule formation in *D. glomerata* has not been studied in detail since the microsymbiont is not cultivable; without a quantifiable inoculum, time course analysis is difficult. At any rate, *D. glomerata* nodule primordia are induced in the root pericycle and lead to the formation of nodule lobes with a central vascular system, while legume nodule primordia are induced in the root cortex and, in case of L*. japonicus,* lead to the formation of a determinate nodule without persistent meristem. Since our study focuses on the induction of *NIN* expression and does not touch on nodule organogenesis, we mention only the differences in the location of the nodule primordium in *D. glomerata vs. L. japonicus* roots.

Reviewer #5 comment 4:

Conclusion
Usually, the conclusion is the key part of any manuscript, the authors has provided the refence, there is no need of reference. Conclusion of the present must be supported by strong statements justifying and summarizing the results and need to add future prospective.

Response to #5-4:

We fully rewrote the conclusion, which now reads as:

“Conclusion

An axenic hydroponic system was established to examine the effects of exogenously applied phytohormones on the expression of key genes required for nodule organogenesis on roots of the actinorhizal plant *Datisca glomerata* and on those of the model legume *Lotus japonicus*, which we used as a control. Marker genes for phytohormones were established: *DgARR9* served as marker for the synthetic cytokinin BAP, whilst *DgGH3.1* and *DgSAUR1* served as markers for the synthetic and natural auxins NAA and PAA, respectively. The collected data showed that *DgNIN2*, whose promoter contains a standard *PACE* *cis-*regulatory element, is not expressed in roots, whereas *DgNIN1*, whose promoter harbors a non-standard *PACE*, showed induction by the auxin PAA, but not by the cytokinin BAP. In contrast*, LjNIN* expression was induced by BAP, NAA, and, when measured 24 h after application of 10 nM, by PAA. The induction of *DgCYCLOPS, DgNIN1* and *DgNF-YA1* transcription by PAA was abolished when either BAP, the ethylene biosynthesis inhibitor AVG or the gibberellin biosynthesis inhibitor PZB were added together with PAA, suggesting that the induction by PAA required certain levels of ethylene and gibberellin, but could be abolished by low levels of exogenously applied cytokinin BAP. Altogether, the phytohormone involvement in the induction of nodules in the actinorhizal species *D. glomerata* differs from that of legumes concerning the roles of auxin, cytokinin and ethylene, while the role of gibberellin has been likely conserved across lineages.”

Reviewer #5 comment 5:

References:
Some references are too old, I suggest updating.

Response to #5-5:

With regard to original research papers, we have done our best to cite the oldest one that contains the information we have to refer to. If this is about reviews, we could try to find younger ones – we would however like to point out that our review on actinorhizal plants from 2012 was chosen because it covers *D. glomerata* nodule structure in some detail, and we are not aware of a younger review that does that. We will gladly make an effort to exchange references but could you please point out which ones appear too old to you?

Reviewer #5 comment 6:

Figures: I would strongly suggest replacing the figures with original ones having high resolution.

Response to #5-6:

The figures we submitted are in .tif format and have high resolution. The problem must be due to the process of assembling the manuscript in the editorial manager.

**Reviewer #6:**

In the present work, the author focuses on the regulation of genes encoding key regulators of symbiotic nodules development in the actinorhizal plant Datisca glomerata by the phytohormones phenylacetic acid (PAA) or Benzylaminopurine (BAP). The mechanisms underlying the symbiotic nodule development and its hormonal control are well established for legume-rhizobia symbiosis at the molecular level; however, for actinorhizal plants, its regulation remains poorly understood. In my opinion, the results of this study are interesting and propose that key regulators of symbiosis are differentially regulated in legumes and actinorhizal, in particular, the expression of the DgNIN gene, which is induced by PAA, but not by BAP as it was shown for the model legumes Lotus japonicus. The manuscript was previously reviewed, and in my opinion, the authors addressed the concerns raised by the reviewers very well; now, the results are presented and discussed adequately.
However, minor concerns must be addressed:

Reviewer #6 comment 1:
- I suggest using “possess” instead of “go back to” in the abstract and introduction.

Response to #6-1:

This was changed in both the Abstract and the Introduction of the current version.

Reviewer #6 comment 2:
- The term "soil recovery" is more appropriate than "soil reclamation" in the introduction.

Response to #6-2:

This was changed in the Introduction of the current version.

Reviewer #6 comment 3:
- The foot of Figure 2 is not correctly indicated: A) It refers to the level of transcripts; B) Refers to the PACE Cis-regulatory elements in the promoter of NIN1 and NIN2…
- Reference to the figure 2A is missing in the results section.

Response to #6-3:

A reference to figure 2A is now included:

“…and although both *NIN1* and *NIN2* are induced in nodules compared to roots, only *NIN2* is expressed nodule-specifically with no detectable levels of expression on roots of either seedlings or greenhouse-grown plants (**Fig 2A;** for *NIN1* see also [55]).”

The figure caption was modified as follows:

“Fig 2 | The *PACE* elements of *DgNIN1* and *DgNIN2* put in context. (A) Expression profile of genes encoding orthologs of nuclear transcription factors associated with nodule development in *Datisca glomerata*; transcript abundance was analysed by RT-qPCR in roots (R) and nodules (N) of greenhouse-grown plants and is given relative to that of the housekeeping gene *EF1-*α (n=3 for both technical and biological replicates); differences between R and N are shown at p<0.001 (student’s *t* test). (B) *In silico* analysis of the *cis-*regulatory element *PACE* in *DgNIN1* and *DgNIN2* promoters; representative logo profile of *PACE* depicted as position weight matrix out of 14 FaFaCuRo species and 2 non-nodulators (*Prunus* *persica* and *Ziziphus jujuba*) along with a comparison of *NIN PACE* sequences in *D. glomerata* showing an 8-nucleotide insertion at the core of *DgNIN1 PACE.* (C) Evaluation of secondary order effects by propeller-twist (ProT) of base-pairs across the *PACE* landscape of *D. glomerata* and model legumes. (D) Implications of primary sequence dissimilarity in secondary structure of CYCLOPS shown as 3D models. Partial alignment of CYCLOPS from 8 nodulating species and 1 non-nodulator (*Trema tomentosa*) shows dissimilarities in a region encompassing 24 residues (see dashed box) (full alignment in **S2A Fig**). (E) AlphaFold 3D models showing the implications in secondary structure of DgCYCLOPS at the site of the region covered by the dashed box, highlighting the presence of an α-helix in DgCYCLOPS, from 402Q to 412D, which is absent in *Ceanothus thyrsiflorus* and model legumes.”

Reviewer #6 comment 4:
- Figure S1 is the same as figure S4. Correct the references to S4 Fig in the results section.
- The S4 Fig footnote does not correspond to S4 Fig. S4 Fig is missing.

Response to #6-4:

We apologize for the error. We have now added the true S4 Fig and deleted the duplicate of S1 Fig.

Reviewer #6 comment 5:
- The S5 Fig footnote is not necessary.

Response to #6-5:

Something must have gone wrong during the previous submission; we apologize. We have included the complete S5 Fig now.
